# Neural correlates of weighted reward prediction error during reinforcement learning classify response to cognitive behavioral therapy in depression

**DOI:** 10.1126/sciadv.aav4962

**Published:** 2019-07-31

**Authors:** Filippo Queirazza, Elsa Fouragnan, J. Douglas Steele, Jonathan Cavanagh, Marios G. Philiastides

**Affiliations:** 1 Institute of Neuroscience and Psychology, University of Glasgow, Glasgow, UK.; 2 School of Psychology, University of Plymouth, Plymouth, UK.; 3 Division of Imaging Science and Technology, University of Dundee, Dundee, UK.; 4 Sackler Centre for Psychobiological Research, Institute of Health and Wellbeing, University of Glasgow, Glasgow, UK.

## Abstract

While cognitive behavioral therapy (CBT) is an effective treatment for major depressive disorder, only up to 45% of depressed patients will respond to it. At present, there is no clinically viable neuroimaging predictor of CBT response. Notably, the lack of a mechanistic understanding of treatment response has hindered identification of predictive biomarkers. To obtain mechanistically meaningful fMRI predictors of CBT response, we capitalize on pretreatment neural activity encoding a weighted reward prediction error (RPE), which is implicated in the acquisition and processing of feedback information during probabilistic learning. Using a conventional mass-univariate fMRI analysis, we demonstrate that, at the group level, responders exhibit greater pretreatment neural activity encoding a weighted RPE in the right striatum and right amygdala. Crucially, using multivariate methods, we show that this activity offers significant out-of-sample classification of treatment response. Our findings support the feasibility and validity of neurocomputational approaches to treatment prediction in psychiatry.

## Teaser

fMRI activity encoding acquisition and processing of feedback enables discrimination of response to self-help CBT in depression.

## INTRODUCTION

Major depressive disorder (MDD) is a common and disabling mental illness (*1*). Cognitive behavioral therapy (CBT) is an effective evidence-based intervention for MDD (*2*). In the United Kingdom, computerized CBT (cCBT) (that is, internet-delivered self-help CBT) is recommended as a first-line treatment option for mild to moderate depression in the National Institute for Health and Care Excellence guidelines. Yet, only up to 45% patients will respond to CBT (*3*).

Despite multidisciplinary efforts (*4*–*10*), the discovery of a reliable and clinically viable predictor of treatment response remains elusive in depression and, in general, in psychiatry (*11*). Notably, previous imaging work has documented that lower pretreatment activity in the subgenual (*4*, *5*) and rostral (*9*) anterior cingulate cortex and higher pretreatment activity in the amygdala (*5*) are associated with response to CBT. Nonetheless, neuroimaging techniques have yet to be adopted as routine clinical diagnostic and prognostic tools in psychiatry (*12*). Multivariate classification of brain imaging data can inform the discovery of neural signatures of treatment response in MDD (*13*). First, multivariate classifiers can yield predictions at the individual level. Second, they can be tuned to the fine-grained and spatially distributed information patterns encoded in large-scale brain activity and may thus be more sensitive in identifying neural biomarkers than conventional imaging analysis methods (*14*). A handful of studies have already pioneered the application of multivariate classification approaches to neuroimaging data to predict treatment response in MDD with encouraging results (*10*, *15*–*17*). Nonetheless, multivariate classifiers remain agnostic about the mechanisms underpinning treatment response. This lack of mechanistic interpretability hinders our understanding of disease processes such as response to treatment. Crucially, it does not provide any insight into previously unidentified targets for drug development. A so-called generative-embedding approach affords a solution to this problem (*18*). First, a generative model describing the hidden mechanisms of specific disease processes is used to extract theoretically meaningful features from a high-dimensional dataset (such as those in brain imaging studies). Second, the model-based feature space is fed into a classification algorithm to make predictions of interest on individual subjects.

The choice of a relevant generative model in MDD is dictated by a wealth of behavioral and neural findings, suggesting that learning from positive (reward) and negative (punishment) feedback [also known as reinforcement learning (RL)] is substantially impaired in depressed subjects (*19*). The computational backdrop of RL affords a formal and normative account of the process of learning from feedback information. Briefly, learning is cast as an optimization problem whereby, while positive outcomes are maximized, negative outcomes are minimized. In reward paradigms, RL is driven by the phasic activity of dopamine neurons in the midbrain, signaling a temporal difference reward prediction error (RPE) (*20*). There is already compelling evidence indicating that this RPE signal is disrupted in MDD. Previous seminal studies using functional magnetic resonance imaging (fMRI) have shown blunting of the RPE signal in the striatum of depressed subjects (*21*–*23*).

While the RPE supports the acquisition of previously unknown information about environmental stimuli, the processing of this information is governed by a weighting parameter known as the learning rate. Crucially, the role of an adaptive learning rate is to adjust the updating of expectations based on environmental uncertainty (that is, the more stable the environment, the lower the learning rate). In probabilistic reward learning paradigms mimicking real-life, volatile environments, a dynamic (that is, time varying) learning rate has been shown to predict choices more accurately than a constant learning rate (*24*, *25*). Thus, an RPE weighted by a dynamic learning rate (which in Bayesian settings has also been referred to as precision-weighted prediction error) encodes both the acquisition and processing of feedback information and represents the process of updating prior expectations (or knowledge) in its entirety.

CBT was developed by Beck (*26*) as a treatment for MDD based on the empirical observation that biased acquisition and processing of feedback information gives rise to and perpetuates depressive symptoms. Accordingly, the clinical practice of CBT in MDD primarily involves identifying and subsequently correcting negatively biased and inaccurate inferences drawn from probabilistic feedback. In other words, this process of cognitive restructuring relies on the patient’s ability to modify (that is, update) negatively biased beliefs (that is, expectations) regarding the self, the world, and the future (also known as the cognitive triad) in the face of revised evidence in favor of or against these beliefs. We propose that, in the computational framework of RL, this cognitive mechanism is captured by a weighted RPE. Only emotion-eliciting (*4*, *5*, *8*–*10*) or task-free resting-state paradigms (*6*, *27*, *28*) have so far been used to probe pretreatment fMRI predictors of CBT response. Moreover, no previous imaging study has attempted to provide a quantitative account of the neural mechanisms underpinning CBT response.

In this study, we hypothesize that the strength of pretreatment neural encoding of the weighted RPE may account for differential response to cCBT in MDD, and using multivariate classification methods, we capitalize on this signal to predict individual response to cCBT. First, we use a computational RL model describing the dynamic updating of expectations during a probabilistic reward-learning task to probe between group differences in blood-oxygen-level-dependent (BOLD) activity associated with model-derived estimates of the weighted RPE and show that BOLD activity in a group of regions, including the right striatum and right amygdala, is significantly greater in the responders group.

To further corroborate clinical utility of our findings and address the potential issue of ecological fallacy (*29*), we also investigate the extent to which pretreatment neural signatures of the weighted RPE could lead to reliable out-of-sample (i.e., individual subject) treatment response classification. Specifically, we perform multivariate classification of BOLD activity encoding the weighted RPE and demonstrate that the same brain regions associated with group-level differences significantly classify individual response to treatment. We show that theory-driven selection of fMRI features yields a good probabilistic estimate of magnitude of symptomatic change at follow-up. Furthermore, we demonstrate that BOLD activity in the right amygdala affords greater discriminative power than activity in the right striatum. Crucially, our findings implicate that neural activity elicited by the weighted RPE during an RL paradigm holds the potential to be adopted as a predictive biomarker of response to cCBT in MDD.

## RESULTS

### Over half of participants responded to internet-delivered self-help CBT

In total, we recruited 37 participants (18 women) via self-referral through local newspaper advertisements. Main eligibility criteria were a primary diagnosis of depressive disorder as operationalized by International Classification of Diseases (10th Revision) diagnostic criteria and a score of ≥14 on the Beck’s Depression Inventory-II (BDI-II) (*30*). To avoid any potential confound associated with psychotropic medications, we only recruited unmedicated depressed subjects.

Participants attended two appointments (before and 2 months after completion of cCBT). Following the first appointment, participants engaged in an online CBT-based guided self-help program, “Living Life to the Full Interactive” (http://llttf.com), which was developed at the University of Glasgow and was designed to help people learn, understand, and overcome their difficulties. Each appointment included a clinical evaluation by a qualified psychiatrist, followed by an fMRI scan. A clinical diagnosis of depression was corroborated using the Clinical Interview Schedule-Revised (CIS-R) (*31*). To measure depression severity and evaluate treatment response, we used the BDI-II, which is a clinically validated tool to assess intensity of depression (*30*).

**Fig. 1:**
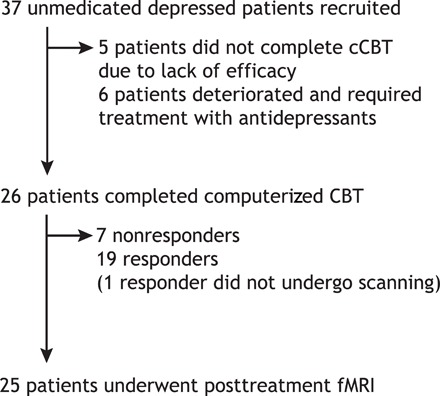
CONSORT (Consolidated Standards of Reporting Trials) diagram for patients in the study.

Eleven subjects (~29.7%) did not attend the posttreatment assessment and did not complete cCBT. Of these subjects, six deteriorated and required treatment with antidepressant medications and five did not complete cCBT due to lack of efficacy. High dropout rates are common in studies examining internet-delivered psychotherapies (*7*). Twenty-six subjects (~70.3%) attended the posttreatment appointment and completed all six modules of our CBT-based intervention. Of these subjects, only one was unable to undergo scanning. In total, 19 subjects were classified as responders and 18 subjects were classified as nonresponders (Fig. 1). The overall cCBT response rate was 51.3%. The average posttreatment improvement in BDI-II score was around 62% (±40%).

Demographic and clinical characteristics of our sample are shown in Table 1. Between-group comparisons revealed no significant differences in age (*t*_35_ = 0.08, *P* = 0.93) and sex (χ^2^_1_ = 0.02, *P* = 0.86). Nonresponders had a significantly higher pretreatment BDI-II score than responders (*t*_35_ = 2.86, *P* = 0.006). On further sensitivity analyses, we found this significant difference to be mainly determined by those nonresponders who did not complete cCBT (*t*_27.3_ = 4.74, *P* < 0.001) rather than by those who did (*t*_24_ = 0.62, *P* = 0.53).

**Table 1:** Demographic and clinical characteristics of the sample. Means and SD (in parentheses) are shown.

	**Responders (*n* = 19)**	**Nonresponders (all) (*n* = 18)**	**Nonresponders (dropouts) (*n* = 11)**	**Nonresponders (retained) (*n* = 7)**
Age (years)	38.99 (12.03)	39.34 (13.43)	35.51 (14.33)	45.35 (9.99)
Gender (male/female)	9/10	10/8	6/5	4/3
BDI baseline	23.89 (8.99)	31.94 (8.01)	35.45 (4.27)	26.42 (9.67)
BDI follow-up	4 (3.43)	Not available	Not available	24.28 (12.48)

### Behavioral performance during probabilistic RL does not discriminate response to cCBT

**Fig. 2:**
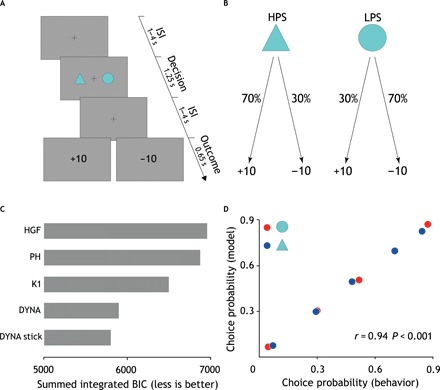
Probabilistic reversal-learning task, computational model comparison, and behavioral fit. (**A**) Each trial commenced with a jittered interstimulus interval (1 to 4 s) displaying a fixation cross. Subsequent to this, two abstract visual stimuli appeared randomly on either side of the screen for 1.25 s. For each participant, the two stimuli were randomly chosen from a pool of 18 different geometrical shapes. Participants were given 1 s to choose a stimulus via a button press. Following a second jittered interstimulus interval (1 to 4 s), participants were presented with the outcome of their decision for 0.65 s. Outcome was either positive (+10) or negative (−10). To maximize design efficiency, the duration of jittered interstimulus intervals was optimized implementing a genetic algorithm. ISI, interstimulus interval. (**B**) Stimulus-outcome contingencies were asymmetrically skewed (70 to 30%) so that the expected value of the two stimuli was of the same magnitude but of opposite sign. This meant that while one stimulus [here referred to as the high-probability stimulus (HPS)] was associated with a greater likelihood of positive outcome, the other stimulus [here referred to as the low-probability stimulus (LPS)] was associated with a greater likelihood of negative outcome. Reversals were self-paced and occurred when participants chose the high-probability stimulus five times over the last six trials. To prevent participants from figuring out the underlying reversal rule, we ran a randomly generated number of buffer trials from a zero-truncated Poisson distribution before reversing stimulus-outcome contingencies. The stimulus-outcome association strength was chosen to enable detection of reversals. (**C**) Summed integrated Bayesian Information Criterion (BIC_int_) scores for all models. Lower scores indicate better fit. DYNA stick is the winning model (BIC_int_ = 5799). DYNA, Krugel *et al.*’s model (BIC_int_ = 5897); DYNA stick, Krugel *et al.*’s model with additional choice autocorrelation parameter stick; HGF, hierarchical Gaussian filter (BIC_int_ = 6957); PH, Pearce-Hall (BIC_int_ = 6875); K1, Kalman filter K1 variant (BIC_int_ = 6498). (**D**) Scatterplot showing linear correlation between the empirical and predicted choice probabilities. *r*, Pearson’s correlation coefficient; *P*, *P* value.

To probe the neural correlates of probabilistic RL, we used a probabilistic reversal-learning task during fMRI (Fig. 2, A and B). This task involved learning which of two stimuli yielded the highest payoff rate and included 180 trials lasting approximately 20 min. Stimulus-outcome contingencies were probabilistic and asymmetrically skewed (70 to 30%; Fig. 2, A and B). Furthermore, stimulus-outcome contingencies were reversed when participants chose the high-probability stimulus (that is, the stimulus with a greater chance of yielding a positive outcome) five times over the last six trials (see Materials and Methods for further details).

Using a binomial test to compare the number of correct choices with chance level, we found that none of the participants chose randomly (binomial test, *P* = 0.5, *P* < 0.05) during the task. We regressed subject-wise percentage of high-probability stimulus choices (as an index of choice accuracy) on clinical outcome after adjusting for the pretreatment BDI-II score and did not find any between-group difference in task performance (*t*_34_ = −0.61, *P* = 0.54). Nonsignificant results were also obtained when noncompleters were excluded from the analysis (*t*_23_ = −0.19, *P* = 0.84) or when only noncompleters were retained as nonresponders (*t*_27_ = −0.81, *P* = 0.42).

In addition, using a generalized mixed-effects linear model, we did not find any significant between-group difference in the effect of the high-probability stimulus on choice behavior (the higher the effect of the high-probability stimulus, the more optimal the choice behavior and thus the learning) (*t*_35_ = −0.24, *P* = 0.80). Likewise, no significant difference was found in subsequent sensitivity analyses (noncompleters removed from nonresponders: *t*_24_ = −0.13, *P* = 0.89; only noncompleters retained as nonresponders: *t*_28_ = −0.20, *P* = 0.83). In summary, pretreatment behavioral measures indexing optimality of choice behavior were not significantly associated with treatment response.

### Surprise drives processing of probabilistic feedback

We fitted five different models (see Materials and Methods for further details) that use trial-wise scaling of the RPE (or equivalent of) but make different assumptions on the computational mechanisms supporting this dynamic tuning of learning rate. On formal Bayesian model comparison (see Materials and Methods for further details), we found that the best-fitting model was that described in (*24*) (Fig. 2C). This model reprises Pearce-Hall’s theory that surprise (formalized as the unsigned prediction error) drives the acquisition of stochastic stimulus-outcome contingencies (*32*) but with some important refinements. Compared to the Pearce-Hall’s model, the smoothing of the unsigned prediction error (the degree of which is regulated by a free parameter ρ) should render the inference process about whether a change has occurred in the environment more robust to the inherent task stochasticity. Moreover, an additional free parameter γ controls the extent to which the dynamic updating of the learning rate is influenced by the slope. For example, while lower values of γ yield substantial trial-by-trial changes of the dynamic learning rate even in the presence of low slope estimates (that is, low surprise), higher values of γ result in a more stable learning rate even in the presence of high slope estimates (that is, high surprise). Hence, this model also allows for the possibility that subjects might be using a relatively fixed learning rate. The decision function was a standard sigmoid function parameterized by the inverse of the temperature parameter (β) reflecting degree of choice stochasticity and by a parameter stick indicating individual choice autocorrelation.

We verified the model’s goodness of fit using a binomial test and found that, under the null hypothesis that on each trial the model was choosing at chance level, the probability of model’s correctly predicted n choices was <0.05 across all subjects. Moreover, using Pearson’s correlation coefficient, we found that observed and model’s predicted choice probabilities were significantly correlated (*r* = 0.94, *P* < 0.001) and that the model’s average predictive probability was 0.67, further endorsing the quality of the model fits (Fig. 2D). Last, using multiple regression, we found that the linear combination of the model’s parameters explained subjects’ behavior (i.e., choice accuracy) well (*R*^2^ = 0.41, *F*_5,37_ = 5.62, *P* = 0.001).

### Responders show greater smoothing over previous unsigned prediction errors than nonresponders

**Fig. 3:**
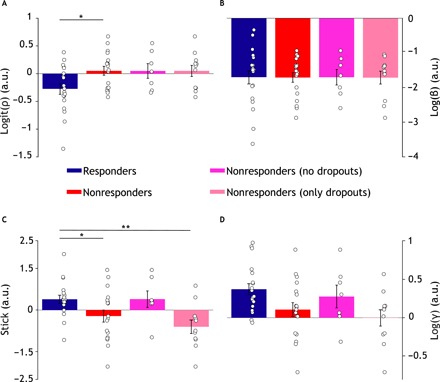
Computational parameters. Bar plots showing computational model parameter estimates. Mean estimates ± SEM (error bars) of 𝜌 (**A**), β (**B**), stick (**C**), and γ (**D**) in the responders (blue; *n* = 19), nonresponders (red; *n* = 18), nonresponders noncompleters (fuchsia; *n* = 7), and nonresponders completers (magenta; *n* = 11) groups. Parameter estimates are shown in their native space (logit for 𝜌 and log for 𝛾 and β). White circles represent individual subjects. *P < 0.05 and **P < 0.001. a.u., arbitrary units.

As we regressed treatment response against computational model’s free parameter estimates (in their native space) after adjusting for the pretreatment BDI-II score, we found a statistically significant between-group difference in the estimates of the model’s parameter ρ [logit(ρ): *t*_34_ = −2.18, *P* = 0.035] and stick (*t*_34_ = 2.08, *P* = 0.045) but no significant difference for β [log(β): *t*_34_ = −0.08, *P* = 0.97] and γ [log(γ): *t*_34_ = 1.48, *P* = 0.14]. Additional sensitivity analyses are as follows: (i) responders versus nonresponders noncompleters [logit(ρ): *t*_27_ = −1.62, *P* = 0.11; stick: *t*_27_ = 6.36, *P* < 0.001; log(β): *t*_34_ = −0.26, *P* = 0.79; log(γ): *t*_27_ = −1.77, *P* = 0.08] and (ii) responders versus nonresponders completers [logit(ρ): *t*_23_ = −1.61, *P* = 0.12; stick: *t*_23_ = −0.12, *P* = 0.90; log(β): *t*_34_ = 0.07, *P* = 0.94; log(γ): *t*_27_ = 0.46, *P* = 0.64] (Fig. 3).

We found that a logistic regression model of clinical response including the model’s parameters and pretreatment BDI-II score significantly improved this model’s fit over and beyond a model including BDI-II only (likelihood ratio test, χ^2^_4_ = 11, *P* = 0.026). Both pretreatment BDI-II (*t*_31_ = −2.22, *P* = 0.025) and the model’s parameter ρ [logit(ρ); *t*_31_ = −2.06, *P* = 0.038) significantly contributed to the prediction of treatment response.

Together, these results show that cCBT responders were, on average, more prone (that is, lower mean estimate of ρ) to smoothing over previous unsigned prediction errors than nonresponders. This implies that responders took greater account of previous feedback history than nonresponders or, alternatively, that nonresponders retrospectively discounted previous reward history more than responders. We found that computational assays significantly improve prediction of treatment response over and beyond routinely collected clinical variables.

### Responders exhibit greater pretreatment BOLD activity encoding the weighted RPE in the right striatum and amygdala

**Fig. 4:**
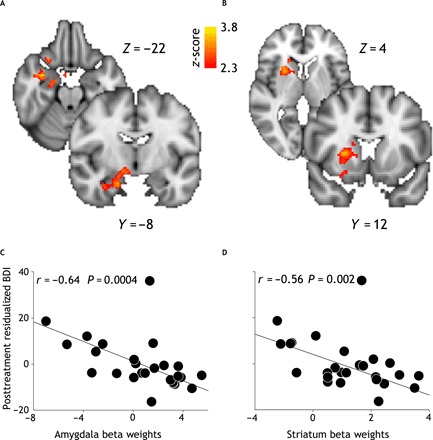
Univariate fMRI analysis. Top: Contrast image representing between-group differences (responders > nonresponders) of neural encoding of the weighted RPE (*P* < 0.05 FWE). Responders exhibit greater activity in the right amygdala (**A**) and right striatum (**B**). Coordinates are given in the MNI (Montreal Neurological Institute) space. Bottom: Scatterplots [*n* = 26 (19 responders and 7 nonresponders)] representing robust linear correlation between posttreatment residualized BDI scores (that is, adjusted for the pretreatment BDI score) and subject-specific average parameter estimates extracted from two clusters pertaining to the right amygdala (**C**) and right striatum (**D**).

A two-sample unpaired *t* test of weighted RPE contrast images revealed significantly greater pretreatment BOLD activity in the responders group in a cluster including the right amygdala extending into the hippocampus (Fig. 4A) and in a cluster including the right caudate and putamen (Fig. 4B). Significant activations survived family-wise error (FWE) correction for multiple comparisons (*P* < 0.05) at the cluster level with a cluster-defining threshold of *P* < 0.01.

Using robust 20% bend correlation, we tested for significant correlations between subject- and cluster-wise average beta weights and magnitude of clinical improvement at 3 months follow-up (as indexed by the residualized follow-up BDI-II score). We found a significant negative correlation between activity in the cluster including the right amygdala and hippocampus and residualized BDI-II at follow-up (*r* = −0.64, *P* < 0.001) (Fig. 4C). Correspondingly, we found a significant correlation between activity in the cluster including the right putamen and caudate and residualized BDI-II at follow-up (*r* = −0.56, *P* = 0.002) (Fig. 4D).

These findings suggest that pretreatment neural activity supporting acquisition and processing of feedback information during RL is comparatively greater in the responders group and scales with the magnitude of posttreatment symptomatic change.

### Pretreatment activity encoding the weighted RPE yields significant binary and probabilistic classification of individual response to cCBT

**Fig. 5:**
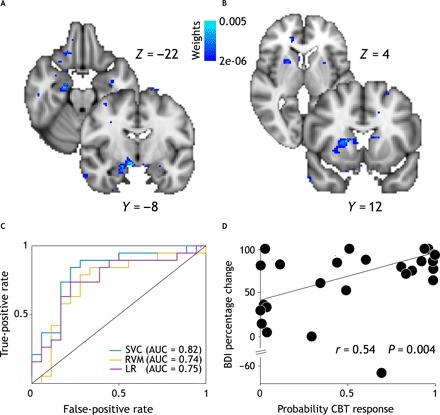
Multivariate fMRI analysis. Top: Weight maps obtained performing feature selection. The right amygdala (**A**) and right striatum (**B**) were the most discriminative features. Coronal and axial slices shown here are same as for univariate analysis (see Fig. 4) to allow for direct comparison between multi- and univariate analyses. (**C**) Receiver operating characteristic curves [and respective areas under the curve (AUCs)] for L2-loss and L2-regularized support vector classifier (SVC; blue), L2-regularized logistic regression (LR; purple), and relevance vector machine (RVM; yellow). We used a leave-one-subject-out nested cross-validation scheme and performed hyperparameter tuning using Nelder-Mead optimization routine. (**D**) Scatterplot showing significant robust linear correlations between subject-wise likelihood of treatment response as estimated by RVM classifier and posttreatment BDI percentage change.

To test whether the observed between-group differences based on group-level fMRI analysis generalize to individual classification of treatment response, we performed multivariate classification of spatially normalized whole-brain contrast images encoding the weighted RPE. Using a leave-one-subject-out nested cross-validation scheme, we found that a linear support vector classifier yields a balanced classification accuracy of 71.57% [95% confidence interval (95% CI), 57.22 to 83.97; sensitivity, 63.16%; specificity, 83.33%; positive predictive value (PPV), 80%; negative predictive value (NPV), 68%] with an area under the curve (AUC) of 0.82 (95% CI, 0.66 to 0.95) and outperforms logistic regression (balanced accuracy, 69.5%; 95% CI, 54.82 to 82.28; sensitivity, 57.89%; specificity, 83.33%; PPV, 79%; NPV, 65%; AUC = 0.75; 95% CI, 0.57 to 0.90) (Fig. 5C). Furthermore, using binomial test, we found classification accuracy to be significantly better than chance (*P* = 0.0025). Crucially, when we implemented a feature selection step using logistic regression with elastic net penalty, we found two clusters in the right amygdala and right striatum to be the most discriminative features of treatment response at the individual level. These clusters largely overlap with those obtained from between-group comparisons as shown in Fig. 5, A and B, providing evidence that the between-group differences observed in the group-level fMRI analysis generalize to the individual. The support vector classifier including the feature selection step (AUC = 0.70; 95% CI, 0.52 to 0.86) does not outperform the support vector classifier without the feature selection step (AUC = 0.82; 95% CI, 0.66 to 0.95), although the difference is not statistically significant (*D* = 1.49, *P* = 0.13, 2000 bootstrap replicates). The classification performance of a classifier depends on the multivariate properties (i.e., correlated signal and noise) of its set of features, and it is thus possible that those features excluded by the feature selection step may be improving overall classification performance by enhancing signal and/or suppressing noise in the selected features.

While so far, we have relied on binary classification of treatment response (i.e., response versus nonresponse), a probabilistic output quantifying uncertainty in this outcome may better inform clinical decision-making especially in the presence of a partial response. Using a probabilistic classifier such as the relevance vector machine (RVM; shown in fig. 5C), we found that estimates of individual likelihood of treatment response are significantly correlated with posttreatment percentage improvement in BDI-II scores (*r* = 0.53, *P* = 0.004) (Fig. 5D). This finding extends the results obtained from the support vector classifier and suggests that BOLD activity encoding the weighted RPE is crucial to both categorical and continuous classifications of cCBT response. The RVM classifier achieved a balanced accuracy of 68.33% (95% CI, 53.77 to 81.38; *P* = 0.006; sensitivity, 68.42%; specificity, 72.22%; PPV, 72%; NPV, 68%) and an AUC of 0.73 (95% CI, 0.55 to 0.90).

### BOLD activity in the right amygdala yields greater classification performance than activity in the striatum

In a supplementary analysis motivated by our goal to unpack the relative contribution of the right amygdala and striatum to the classification of treatment response, we compared the AUC of two models including cluster-wise beta weights from either the right amygdala or right striatum. We show that the right amygdala (AUC = 0.83; 95% CI, 0.68 to 0.95) has significantly greater discriminative power than the right striatum (AUC = 0.62; 95% CI, 0.43 to 0.81; *D* = 2.09; *P* = 0.03; 2000 bootstrap replicates). Although both the right amygdala and right striatum are selected during the feature selection step, it is possible that selection of the right striatum improves classification performance of the elastic net classifier by enhancing signal and/or suppressing noise in the right amygdala. To validate the clinical utility of model-based fMRI features as predictive biomarkers of response to CBT in MDD, we compared the classification performance (as indexed by the AUC) of two predictive models (pretreatment BDI-II scores only versus right amygdala beta weights plus pretreatment BDI-II scores). We found that the model including fMRI features (AUC = 0.83; 95% CI, 0.68 to 0.98) exceeds the classification accuracy of the model including pretreatment BDI-II scores only (AUC = 0.75; 95% CI, 0.57 to 0.90) although not significantly (*D* = −0.82, *P* = 0.20, 2000 bootstrap replicates).

## MATERIALS AND METHODS

### Experimental design and paradigm

We adopted a naturalistic longitudinal design. Participants attended two appointments (before and 2 months after treatment with cCBT). Each appointment included a clinical evaluation by a qualified psychiatrist, followed by an fMRI scan. A clinical diagnosis of depression was corroborated using the CIS-R (*31*). We chose to evaluate clinical outcome using the BDI-II, since CBT mainly targets the cognitive symptoms of depression and the BDI-II focuses on the cognitive rather than biological symptoms of depression.

Inclusion criteria were a primary diagnosis of depressive disorder as operationalized by ICD-10 diagnostic criteria and a score of ≥14 on the BDI-II (*30*). Exclusion criteria included current prescription of psychotropic medications, current involvement with other CBT-based interventions or psychological therapies, a comorbid diagnosis of other major mental disorders, CBT treatment within the past 3 years, a diagnosis of psychoactive substance dependence, and previous history of brain injury.

We regarded noncompletion of cCBT as an index of treatment failure and classified all noncompleters as nonresponders. For the remaining subjects, response to cCBT was defined as a 50% or greater reduction in the pretreatment BDI-II score. To account for response bias, we performed sensitivity analyses excluding noncompleters from the nonresponders group.

We used a probabilistic reversal-learning task during fMRI (Fig. 2A). This task involved learning which of two stimuli yielded the highest payoff rate and included 180 trials lasting approximately 20 min. Participants were required to choose between two abstract visual stimuli both yielding either a positive (+10) or negative (−10) outcome devoid of monetary value. Stimulus-outcome contingencies were probabilistic and asymmetrically skewed (70 to 30%; Fig. 2B). Furthermore, to create a volatile environment, stimulus-outcome contingencies were reversed in the course of the experiment. Notably, reversals were triggered when participants chose the high-probability stimulus (that is, the stimulus with a greater chance of yielding a positive outcome) five times over the last six trials. As a result, participants experienced a different number of reversals. Participants were advised of the probabilistic nature of the task and that stimulus-outcome contingencies might reverse based on their performance. Moreover, to ensure that participants understood the nature of the task, they underwent a 5-min practice session before the fMRI scan. The task was programmed using Presentation (Neurobehavioural Systems) stimulus delivery software. The probabilistic reversal-learning task is ideally suited to exposing biases in acquisition and processing of feedback during probabilistic learning. Ultimately, optimal performance rests on the subjects’ ability to infer whether fluctuations in observed stimulus-outcome associations reflect either noise (given underlying stochastic contingencies) or sudden environmental changes (that is, reversals). All participants provided written informed consent. The study protocol was approved by the West of Scotland Ethics Committee (10/S0703/71).

### Computerized CBT

The clinical intervention used in this study consisted of an online CBT-based guided self-help program, “Living Life to the Full Interactive” (http://llttf.com), developed at the University of Glasgow and designed to help people learn, understand, and overcome their difficulties. This intervention was made freely available to participants by Action on Depression, a Scottish depression charity. Participants were required to work through six online modules (each taking around 2 hours to complete) over a period approximately between 6 and 10 weeks and received weekly telephone support from an Action on Depression worker.

The advantage of using self-help CBT is twofold. First, it substantially reduces the confounding effect of therapist variability on treatment response, and second, it permits monitoring of treatment adherence defined as the number of online modules completed. To evaluate sustained response to cCBT and thus account for the placebo effect, participants were reassessed 2 months after completion of cCBT.

### Behavioral statistical analysis

To verify that individual performance was better than chance, we used a binomial test to compare the number of correct choices with chance level. To examine for between-group differences in task performance, we regressed subject-wise percentage of high-probability stimulus choices on clinical outcome after adjusting for the pretreatment BDI-II score. In addition, we tested for any between-group difference in the effect of the high-probability stimulus on choice behavior (the higher the effect of the high-probability stimulus, the more optimal the choice behavior and thus the learning). We first run the following maximal by-subject random intercept and random slope generalized mixed-effects linear model (*33*) using the lme4 package in R (www.r-project.org)

$${\text{choice}}_{\mathrm{ji}}=\mu +\mu_{j}+(\beta_{1}+\beta_{1j})\;\text{hps}+\beta_{2}{\text{BDI}}_{\text{pre}}+\varepsilon_{\mathrm{ji}}$$

where subscripts *i* and *j* refer to the *i*th trial for the *j*th subject, respectively, choice is a two-level factor indexing choice (*a* or *b*), μ is the grand mean, μ*_j_* is the by-subject random intercept, β*_j_* is the by-subject random slope, β is the population-level fixed-effect, hps is a two-level factor indexing the high-probability stimulus (*a* or *b*), BDI_pre_ is a nuisance covariate encoding pretreatment BDI-II score, and ε is the error term. We subsequently retrieved the by-subject random slopes encoding the effect of the high-probability stimulus on choice behavior and regressed them against clinical outcome. To account for potential influential and/or outlying observations, we performed this latter regression analysis using a robust fitting procedure as implemented in the rlm function in R (www.r-project.org).

### Computational modeling of behavioral data

We fitted four different models that use trial-wise scaling of the prediction error. The first model implemented in this paper is the hierarchical Gaussian filter (HGF) (*25*). This model is structured as a three-level hierarchy of random walks coupled by their variances. The categorical binary input *u* ϵ [0,1] represents the association between a specific outcome given a specific choice and was coded in the contingency space as follows

(+10∣HPS)=(−10∣LPS)=1;(−10∣HPS)=(+10∣LPS)=0

The hidden state at the bottom level *x*_1_ is also binary and, given the absence of sensory uncertainty in our task, was assumed to be equal to *u*. Its probability distribution is modeled using a Bernoulli distribution

$$p({x_{1}}^{t}|{x_{2}}^{t})=\sigma {\left({x_{2}}^{t}\right)}^{{x_{1}}^{t}}{\left(1-\sigma ({x_{2}}^{t})\right)}^{1-{x_{1}}^{t}}$$

where σ() is a sigmoid function, which means that *x*_1_ is simply the result of a sigmoid transformation of *x*_2_. It should thus be interpreted as the probability that the high-probability stimulus (i.e., HPS) is associated with positive feedback [or, alternatively, the probability that the low-probability stimulus (i.e., LPS) is associated with negative feedback].

At the second level, *x*_2_, which represents the conditional probability of positive feedback given the high-probability stimulus (or, alternatively, the conditional probability of negative feedback given the low-probability stimulus) in logit space, is modeled with a Gaussian distribution

$$p(x_{2}^{t}|x_{2}^{t-1},x_{3}^{t})=N(x_{2}^{t-1},e^{\left(\kappa x_{3}^{t}+\omega \right)})$$

where κ and ω are two constant parameters encoding the phasic $\left(\kappa x_{3}^{t}\right)$ and tonic (ω) uncertainties of *x*_2_ and superscript *t* is the trial index.

Last, at the highest level, *x*_3_ denotes the log volatility of the environment and is modeled as another Gaussian distribution

$$p(x_{3}^{t}|x_{3}^{t-1},\vartheta )=N(x_{3}^{t-1},\vartheta )$$

where ϑ is the topmost variance and is a constant parameter. The posterior density of the model’s hidden states and free parameters given input *u* is obtained by maximizing the log-model evidence using a variational inversion scheme. Briefly, this leads to analytical update equations, which broadly share the following form

$$\mu_{l}^{t+1}=\mu_{l}^{t}+\frac{{\hat{\pi }}_{l-1}^{t}}{\pi_{l}^{t}}\delta_{l-1}^{t}$$

where subscript *l* indexes the hierarchy’s level, μ*_l_* denotes the posterior mean of state *x_l_*, ${\hat{\pi }}_{l-1}^{t}$ is the precision (that is, inverse of variance) of the prediction onto the level below, $\pi_{l}^{t}$ is the posterior precision at the current level, and δ_*l*−1_ is the prediction error from the level below. A detailed illustration and derivation of the HGF update equations is given in (*25*). In our implementation of the HGF model, we found that fixing ω and optimizing κ and ϑ provided the best fit to the data.

In the model, the learning rate scales with the slope of the smoothed unsigned prediction error (i.e., absolute value of prediction error) (*24*) and only updates the value of the chosen stimulus *v* as follows

$$v^{t}=v^{t-1}+\alpha^{t}\delta^{t}$$

where δ*^t^* is the RPE. The initial value of both stimuli was set to 0. The smoothed unsigned prediction error (pemag) is computed as the convex combination of the absolute value of the prediction error (∣δ|) on trial *t* and of pemag on trial *t* − 1

$${\text{pemag}}^{t}=\rho \;|\delta^{t}|+(1-\rho ){\text{pemag}}^{t-1}$$

where ρ is a free parameter that controls the degree of smoothing over previous unsigned prediction errors. Lower values of ρ denote greater smoothing and thus imply lesser sensitivity to task’s stochasticity.

The normalized slope *m* is computed as follows

$$m^{t}=\frac{{\text{pemag}}^{t}-{\text{pemag}}^{t-1}}{({\text{pemag}}^{t}+{\text{pemag}}^{t-1})/2}$$

Crucially, the slope is a signed quantity, which determines the direction of the learning rate updating. While a positive slope increases the learning rate, a negative slope decreases it

$$\alpha^{t}=\alpha^{t-1}+f(m^{t})(1-\alpha^{t-1}\;),\text{if}\;m^{t}>0$$

$$\alpha^{t}=\alpha^{t-1}+f(m^{t})(\alpha^{t-1}),\text{if}\;m^{t}<0$$

where *f*() is a double sigmoid function that maps the slope to the [0,1] interval. This transformation function is itself parameterized by a free parameter γ, which regulates the steepness of the double sigmoid curve and therefore determines the extent to which each subject is sensitive to surprising feedback information. While lower values of γ denote a greater tendency to update trial-by-trial estimates of the learning rate in the face of surprising incoming information, higher values of γ indicate a relatively surprise-invariant, more stable learning rate. Hence, this model also allows for the possibility that subjects might be using a relatively fixed learning rate. Notably, we also implemented a version of this model where we used a hyperbolic tanh function instead of a double sigmoid function but found that this resulted in a worse model fit.

The third model was an implementation of the Pearce-Hall model where the unsigned prediction error acts as the dynamic learning rate resulting in the following update equations

$$v^{t}=v^{t-1}+S\;\alpha^{t}\delta^{t}$$

where *S* is a constant parameter encoding the intrinsic salience (“associability”) of probabilistic information and α is ∣δ∣. The initial value *v*^1^ was set to 0.5.

The fourth model (K1) used a variant of the Kalman-filter algorithm, as described in (*34*). The codes for the HGF and K1 models were modified from the HGF Toolbox v4.10 freely available at www.tnu.ethz.ch/en/software/tapas.html.

For all above models, the decision function was a standard sigmoid function σ() mapping the difference between the two stimulus expected values *v^t^* to choice propensity *p^t^* as follows: *p^t^* = σ(β*v^t^*) where β is the inverse of the temperature parameter determining stochasticity of the decision function. For the second model, we also used a sigmoid decision function with a parameter stick accounting for the tendency to repeat the same choice.

To preserve the parameters’ natural bounds, log (β, γ) and logit (α, ρ, ϑ) transforms of the parameters were implemented. For the HGF model, we initialized the prior mean and variance of the free parameters as follows: κ (2.2, 16), θ (0.025, 16), and β (1, 1). For κ and θ, we chose an upper bound at 3 and 0.005, respectively. Last, we fixed the value of ω to −4. For the second model described in (*24*), we initialized the free parameters’ (β, γ, ρ, and stick) prior means to (1, 0.5, 0.5, and 0) and their prior variances to 100. For the Pearce-Hall model, we initialized the prior mean and variance of the free parameters as follows: α^1^ (0.5, 1) and S (0.1, 8) and β (1, 16). For the variant K1 of the Kalman filter model, we initialized the prior mean and variance of the free parameters as follows: μ (1, 100), h^1^ (0.005, 16), and β (1, 1).

Hierarchical Bayesian models such as the HGF have been designed to accommodate qualitatively different and hierarchy-dependent sources of uncertainty. However, as already pointed out in (*35*), in an experimental paradigm like ours (i.e., a two-armed bandit where outcome contingencies of one arm can be fully inferred from those of the other arm), tracking different levels of uncertainty is unfeasible. In contrast, the learning rule implemented in Krugel *et al.*’s work (*24*) provides a heuristic that may be more suited to the task used here.

### Model fitting and model comparison

To prevent overfitting, we implemented the hierarchical type II maximum likelihood fitting procedure described in (*36*). For each subject *i*, we found the maximum a posteriori estimate of each model’s free parameters

$$\theta_{i}^{\text{MAP}}={\text{argmax}}_{\theta }\;p(C_{i}|\theta_{i})p(\theta_{i}|\xi )$$

where *p*(*C_i_*∣θ*_i_*) is the cross-entropy loss function between the empirical and predicted choices *C_i_* given the model parameters θ*_i_*, and *p*(θ*_i_*∣ξ) is the prior distribution on the model parameters θ*_i_* given the population-level hyperparameters ξ. To estimate the optimal ξ, we implemented an expectation-maximization algorithm, which performs *k* iterations of a two-stage optimization routine until convergence. Briefly, during the expectation step, we optimized the subject-wise joint distribution over the data and parameters with respect to the parameters holding the hyperparameters fixed

$$\theta_{i}^{\left(k\right)}={\text{argmax}}_{\theta }\;p(C_{i}|\theta_{i})p(\theta_{i}|\xi^{\left(k-1\right)})$$

and found the posterior distribution over the parameters using a Laplace approximation

$$p(\theta |C_{i},\xi^{\left(k-1\right)})=N(\mu ,\Sigma )$$

During the maximization step, we revised the population-level hyperparameters ξ by updating the first and second moments of the multivariate normal distribution over the parameters.

To determine the best-fitting model, we subsequently performed formal Bayesian model comparison among the fitted models using the integrated Bayesian Information Criterion (BIC_int_). We implemented the procedure described in (*36*) and measured each model’s goodness of fit based on the model’s population-level hyperparameters. Here, the model log likelihood was obtained by integrating over the model’s parameters. This integral was approximated by sampling 1000 times the model’s parameters from a Gaussian prior density whose mean and variance are set to the population-level hyperparameters. Each model’s BIC_int_ was then obtained by summing over the individual BIC_int_.

Last, we ran sanity checks on the winning model’s goodness of fit. To verify the accuracy of model’s fit, we first binned predicted choice propensities according to their quintiles and subsequently measured the strength of their linear association with corresponding observed choice probabilities using Pearson’s correlation coefficient. In addition, we used a binomial test to test whether the number of choices correctly predicted by the model exceeded that expected by chance and estimated the model’s predictive probability. To ascertain any between-group differences in the model’s fixed parameters, we regressed subject-wise parameter estimates against clinical outcome (response versus nonresponse) after adjusting for the pretreatment BDI-II score using a robust fit with bisquare error weighting function (robustfit function in MATLAB). To account for the response bias, we performed additional sensitivity analyses without dropouts or with dropouts only. To test the predictive power of the model’s parameters over and beyond the pretreatment clinical score, we performed a likelihood ratio test to compare logistic regression models of treatment response including either model’s parameters plus pretreatment BDI-II scores or pretreatment BDI-II score only as predictors of interest.

### fMRI data acquisition

We used a 3T GE system with an eight-channel parallel imaging head coil. We acquired a high-resolution *T*_1_-weighted structural image (0.5 mm by 0.5 mm by 1 mm voxels, 320 by 320 matrix, 160 axial slices, inversion time (TI) = 500 ms, repetition time (TR) = 7700 ms, echo time (TE) = 1.5 ms, flip angle = 12°) using an optimized inversion recovery fast spoiled gradient echo sequence and a functional echo planar imaging scan (3-mm isotropic voxels, 64 by 64 matrix, 608 axial slices, TR = 2000 ms, TE = 30 ms, flip angle = 80°). Slice orientation was tilted to −20° from the anterior commissure-posterior commissure (AC-PC) plane to alleviate signal dropout in the orbitofrontal cortex. The first four volumes of the functional scan were discarded to allow for the magnetic field to reach the steady state.

### fMRI data preprocessing and mass-univariate statistical analysis

Pretreatment fMRI data preprocessing and statistical analyses were performed using FSL (FMRIB’s software library) software. Preprocessing pipeline involved intramodal motion correction using MCFLIRT (motion correction FMRIB’s linear image registration tool), slice timing correction, spatial smoothing with an isotropic 5-mm full width at half maximum Gaussian kernel, high-pass temporal filtering with a cutoff frequency of 110 s, and grand-mean intensity normalization of each entire four-dimensional dataset. Functional scans were subsequently coregistered with skull-stripped structural images using boundary-based registration as implemented in FLIRT (FMRIB’s linear image registration tool) and spatially normalized into MNI152 space using FNIRT (FMRIB’s non-linear image registration tool) nonlinear registration.

We performed whole-brain statistical analyses of pretreatment fMRI data using a multilevel mixed-effects approach as implemented in FLAME1 (FSL). At the first level for each subject, we built a design matrix modeling both the decision and outcome phases of the behavioral task. For the decision phase, we included the following regressors: (i) two parametric regressors encoding the model-derived trial-wise expected value of chosen and unchosen stimuli and (ii) two nuisance regressors accounting for visual stimulation (unmodulated) and motor response (modulated by reaction time) during the decision phase. For the outcome phase, we created the following regressors: (i) one parametric regressor of interest encoding model-derived trial-wise weighted RPE estimates, (ii) one parametric nuisance regressor representing the magnitude (that is, absolute value) of model-derived RPEs to retrieve the unique effect of the dynamic learning rate on BOLD activity, (iii) two unmodulated nuisance regressors representing positive and negative outcomes (i.e., RPE valence), and (iv) one nuisance regressor modeling lost trials. We included RPE magnitude and valence regressors in our design matrix based on growing evidence that these variables have temporally overlapping but distinct spatial representations in the brain (*37*–*39*). Six additional motion parameters (three translations and three rotations) estimated during the motion correction phase were included as regressors of no interest. We modeled all regressors as boxcar functions. We ensured that our design matrix was well conditioned and not rank deficient using the collinearity diagnostics incorporated in FSL. To improve efficiency of our fMRI statistical analysis, we obtained all model-derived regressors by generating subject-wise model fits using the population-level parameter means. We convolved all regressors with a hemodynamic response function (double gamma function).

We estimated the subject-wise linear contrasts of parameter estimates and subsequently entered these contrast images into a second-level mixed-effects analysis where we tested between-group (responders versus nonresponders) differences using unpaired two-sample *t* test. The design matrix of the second-level analysis also included pretreatment BDI-II as a regressor of no interest. We thresholded the resulting *Z* statistic images using a cluster-defining threshold of *Z* > 2.3 and an FWE-corrected significance threshold of *P* = 0.05. From the second-level contrast image estimating differential BOLD activity (i.e., responders > nonresponders) associated with weighted RPE, we identified two statistically significant clusters. Using these clusters, we then retrieved the cluster-wise average beta weights from the individual contrast images and performed a 20% bend correlation with residualized posttreatment BDI score (i.e., adjusted from the pretreatment BDI score) to test for a significant linear correlation with clinical outcome.

### fMRI multivariate statistical analysis

To classify responders versus nonresponders, we performed multivariate classification of individual spatially normalized contrast images encoding the weighted RPE. With this analysis, we endeavored to address the potential issue of ecological fallacy and test the validity of our group-level fMRI findings as predictive biomarkers at the individual level.

Data preprocessing involved removing nonbrain voxels from spatially normalized contrast images using a standard brain mask and feature-wise (i.e., column-wise) standardization. We used two types of classifiers from the open-source software liblinear: L2-regularized and L2-loss support vector machine and L2-regularized logistic regression (*40*). We chose to use a linear kernel so that each weight represents the strength of the linear association between features and outcome of interest, therefore easing the mechanistic interpretation of a classifier’s weights.

To estimate a classifier’s performance, we implemented a leave-one-subject-out nested cross-validation scheme. Data were randomly partitioned on subjects using the cvpartition function in MATLAB statistical toolbox (www.mathworks.com). While a classifier’s weights were optimized in the outer loop, the hyperparameter C for the L2 penalty term was tuned in the inner loop using fivefold cross-validation. We used a number of optimization routines for hyperparameter C tuning: exhaustive grid search using a built-in function in liblinear (*40*), the derivative-free Nelder-Mead simplex algorithm, random search, and a quasi-random Sobel sequence as implemented in the open-source software library optunity (www.optunity.com). Because of the slight class imbalance (i.e., 19 responders versus 18 nonresponders) during the training phase, we set class weights so that higher misclassification penalties are assigned to training examples from the smaller class. Moreover, to safeguard from biased (that is, overly optimistic) estimates of generalizability, we assessed a classifier’s performance using balanced accuracy and its posterior distribution instead of accuracy, as implemented in (*41*). We also report sensitivity, specificity, PPV, and NPV. In addition, we estimated the area under the receiver operating characteristics curve (AUC) using the trapezoidal rule and its CI using 2000 stratified bootstrap replicates as implemented in the R package pROC (*42*). To assess statistical significance, we used a binomial test and computed the probability of observed classification accuracy under the null hypothesis that the classifier is operating at chance. To assess each voxel’s discriminative ability, we used feature selection before hyperparameter C tuning. We performed feature selection using regularized logistic regression with elastic net penalty term as implemented in the open-source glmnet software package (www.stanford.edu/~hastie/glmnet_matlab/) (*43*). During the feature selection step, we performed tuning of the hyperparameters (alpha and lambda) in the elastic net penalty term using grid search and fivefold cross-validation (for each alpha and lambda pair). Moreover, we optimized model parameters (i.e., regression coefficients) using cyclic coordinate descent. Compared to other regularizers (such as lasso or ridge), the elastic net penalty term has the double advantage of generating a sparse output and of selecting correlated features in and out together (*43*).

Second, to obtain a probabilistic estimate of individual treatment response, we trained a linear kernel RVM classifier, which uses a sparse Bayesian modeling approach and does not require additional regularization parameters (*44*). We used a leave-one-subject-out cross-validation scheme. We retrieved the average subject-wise estimates of the likelihood of treatment response and correlated these with posttreatment BDI-II percentage change using robust 20% bend correlation test. Third, in a supplementary analysis motivated by our goal to unpack the relative contribution of the right amygdala and right striatum to classification of treatment response, we first retrieved the cluster-wise beta weights from individual spatially normalized contrast images and then compared the AUCs of two models (right amygdala versus right striatum) to determine the cluster with the greatest discriminative power. Again, the objective of this supplementary analysis is not to evaluate the absolute value of a classifier’s performance as this is inflated due to issues of circularity (also known as double dipping) but to rank the relative contribution of each cluster to treatment response classification. Last, to evaluate the predictive power afforded by model-based fMRI features over and above pretreatment BDI-II score, we fitted a predictive model using pretreatment BDI-II scores only and tested whether the right amygdala beta weights plus pretreatment BDI-II score significantly exceeded its classification performance. We conducted this analysis by performing pairwise comparisons of the classifiers’ AUCs using the nonparametric bootstrap method (*42*).

## DISCUSSION

In the present study, we address the long-standing issue of developing a reliable and valid predictive biomarker in psychiatry (*11*). Using a generative embedding approach, we focus on theory-driven computational and fMRI assays to develop mechanistically interpretable predictive biomarkers of clinical outcome. Crucially, pathophysiological mechanisms implicated in symptomatic change in MDD are subtle and difficult to uncover. Thus, to enhance the predictive power of our classifiers, we tap into previously well-characterized abnormal cognitive processes in MDD such as learning from probabilistic feedback, which may also be important in the context of clinical response to CBT. More specifically, we examine pretreatment BOLD activity encoding the weighted RPE during RL. At the group level, we demonstrate that responders exhibit greater BOLD activity than nonresponders in the right striatum and right amygdala. Furthermore, we show that BOLD activity in these regions is significantly and linearly correlated with the extent of posttreatment symptomatic change. This latter finding is critical, as it safeguards from any biased inference potentially originating from the number of dropouts in the nonresponders group. Moreover, the clinical significance of this finding is further corroborated by its medium effect size (0.5 ≤ r ≤ 0.8). Of note is our promising finding that parameters embedded in computational models of choice behavior help discriminate treatment outcome. At the individual level, we find that this neural activity significantly classifies response to treatment and yields a probabilistic estimate of posttreatment clinical response, which is significantly and linearly correlated with observed symptomatic improvement. Our classifier is better at identifying false positives than false negatives. In other words, it is better at ruling out patients that would not benefit from cCBT than at ruling in patients that would respond to it. Although, ideally, both specificity and sensitivity should be as high as possible, this is perhaps a more desirable compromise from a clinical standpoint. It does, in fact, mean that patients will be spared from lengthy and ineffective treatments and, most importantly, from their burdensome side effects (*45*) (although of course it also means that some patients will miss out on potentially effective treatments). As a corollary, we provide evidence that activity in the right amygdala has greater discriminative ability than activity in the right striatum and that this activity exceeds (although not significantly) classification accuracy of pretreatment depression severity. We provide evidence that symptom reduction is correlated with decreasing activity in both the right amygdala and right striatum (see Supplementary Materials and Methods and fig. S1).

Only a few studies have, so far, used fMRI to predict response to CBT in MDD (*4*, *5*, *9*, *10*). A major limitation of these studies is the lack of any explicit account of the putative neural mechanisms underpinning CBT response. Crucially, although the focus of CBT in MDD is centered in fostering adaptive reappraisal strategies, to the best of our knowledge, no previous study has examined acquisition and processing of probabilistic feedback as a function of response to CBT. Moreover, despite compelling evidence that behavioral and neural responses to probabilistic feedback are abnormal in depression (*19*), the cognitive mechanisms underlying this impairment are still unknown and relatively unexplored. In this study, we have addressed this knowledge gap by explicitly modeling the neurocomputational mechanisms of inference implicated in probabilistic RL as a function of treatment response. We show that pretreatment neural activity supporting acquisition and processing of probabilistic feedback is comparatively greater in depressed patients who go on to benefit from cCBT. Within the RL framework, enhanced neural encoding of the weighted RPE denotes greater neural updating of expectations regarding the value of environmental stimuli.

Given that the weighted RPE supports learning from probabilistic feedback, a possible interpretation of this finding is that CBT responders may be endowed with greater neural resources to tackle distorted thinking patterns associated with MDD and thus to successfully engage in the work of cognitive restructuring practiced during CBT. Observed relatively greater neural signaling of the weighted RPE in the responders group may facilitate response to CBT by enabling reappraisal strategies and thus by fostering more balanced beliefs about the self and the surrounding world. In other words, responders may be more able to use revised evidence in favor of or against dysfunctional beliefs to modify their thinking patterns. Conversely, relative blunting of the weighted RPE signal may hinder reframing of maladaptive thinking patterns during CBT. This interpretation is in accordance with the capitalization model of the effects of psychological therapies (*46*). According to this model, psychotherapies are successful insofar as they leverage preexisting patients’ strengths.

Alternatively, the compensation model posits that effective psychotherapies remedy disorders relevant to patients’ vulnerabilities (*46*). It cannot be ruled out that our finding may denote aberrant neural activity, which is the therapeutic target of CBT and without which CBT may thus be unable to exert its beneficial effect. Evidence that symptomatic improvement at follow-up correlates with decreasing activity in both the right amygdala and right striatum lends some support to this interpretation. It is possible that comparatively greater neural activity encoding acquisition and processing of feedback information implies a greater propensity to extensively appraise environmental stimuli in the responders group. Heightened information processing may lead to a more pronounced ruminative thinking style, which has already been positively associated with amygdala activity (*5*). In addition, it may cause greater difficulty disengaging from (negative) environmental stimuli, a recognized feature of MDD (*47*). Rumination and negative attentional focus exacerbate negative cognitive biases (*48*), and CBT primarily acts by altering and reversing these cognitive biases.

In our experiment, we use a weighted RPE that conflates signed and unsigned prediction errors. Our dynamic learning rate does, in fact, represent the rate of change of the smoothed unsigned prediction error, which is thought to measure outcome surprisingness. This may account for observed activity in the amygdala, a region that has been previously linked to the encoding of surprise during associative learning in rodents (*49*), nonhuman primates (*50*), and humans (*51*), although a previous study implicates the putamen as well (*52*). In particular, BOLD activity in the amygdala has been shown to covary with trial-by-trial estimates of cue-specific associabilities akin to a dynamic learning rate (*51*). Although, traditionally, the functional role of the amygdala has been linked to threat detection and fear conditioning, there is increasing evidence that the amygdala is also involved in processing attentional relevance of environmental stimuli. Given its extensive projections to sensory pathways and cortical areas, the amygdala has, in fact, the capacity to modulate perceptual, attentional, and cognitive processes to orchestrate appropriate response to environmental stimuli. Furthermore, given its putative role in processing emotional information and widespread connections to the hippocampus, increased amygdala activity may foster consolidation of negatively biased memories in MDD (*53*).

Our finding that neural activity in the right striatum is positively correlated with CBT response is consistent with a previous report that, in a group of adolescents with depression, greater pretreatment striatal responses to both anticipation and presentation of positive feedback during a monetary reward task were linked to posttreatment reduction in depression severity, particularly of anxiety symptoms (*54*). In contrast, another study found that lower pretreatment BOLD response to sad faces in the putamen was associated with greater symptom reduction at follow-up (*9*). However, in this latter study, the different experimental paradigm may account for the observed discrepancy in the results. Greater pretreatment BOLD activity in the right amygdala in association with symptomatic improvement following CBT is also in accord with previous work in this field (*5*). Siegle *et al.* documented that pretreatment (right) amygdala reactivity to negative emotional words was positively correlated with improvement in posttreatment BDI-II scores and high levels of self-reported rumination. Fu *et al.* (*9*) also reported greater right amygdala activity during implicit processing of sad faces in patients with MDD relative to controls; most of these patients (82.5%) responded to CBT and showed reduced right amygdala activity at 4 months follow-up, although a regression-to-the-mean effect could not be ruled out.

Still, increased amygdala activity in response to emotion paradigms may not be specific to prediction of CBT response but may act as a general biomarker of favorable clinical outcome instead. Enhanced amygdala reactivity to emotional visual stimuli has also been linked to better prognosis independent of medication status and symptoms severity (*55*) and to successful treatment with antidepressants (*56*). Moreover, this activity has been reported to normalize with reduction of symptom severity (*56*). Notably, a recent study has highlighted that the within-subject reliability of BOLD activations elicited in the amygdala by emotional faces processing tasks is low, thus implicating that this activity may not constitute a clinically viable biomarker (*57*). In contrast to previous imaging studies that have used emotional paradigms, we do not find pretreatment activity in the perigenual anterior cingulate cortex to be associated with CBT response. Given the role of this brain region in emotion monitoring and generation, it is possible that differences in experimental paradigms (reward-learning tasks versus processing of emotional stimuli) may account for this discrepancy.

In addition to fMRI data, we capitalize on the notion that optimized fixed parameters embedded in our computational model capture a person’s typical mode of appraising incoming probabilistic feedback. We show that responders take greater account of previous feedback history than nonresponders by means of greater smoothing over previous unsigned RPE. This operation helps averaging out noisy feedback from the trial-by-trial online computation of surprise. Thus, it renders inference more robust to random fluctuations in the statistics of the environment and makes the temporal trajectory of the learning rate more stable. Conversely, comparatively lesser smoothing over previous unsigned RPE leads to more erratic updating of beliefs regarding the surrounding environment, which may result in more extreme patterns of thinking.

We speculate that depressed patients who are more adept at thoughtfully sieving through the barrage of noisy feedback information surrounding them may exhibit a greater predisposition to critical thinking. This may translate in a greater ability to challenge maladaptive thinking patterns. In support of this interpretation is the previous finding that pretreatment clinical ratings indicative of lower dysfunctional attitudes (as a result of less rigid and extreme thinking) predict better response to cCBT (*7*).

In recent years, there has been a growing focus on “precision medicine” to improve patient care by tailoring treatments with best outcome and least side-effects burden to each patient. Critical to the implementation of precision medicine is the development of predictive biomarkers of clinical outcome to enable treatment selection and prognostic stratification. Unfortunately, psychiatry is lagging behind other medical disciplines (*12*). Multivariate classification methods applied to fMRI data provide a valuable tool to make predictions of clinical relevance at the individual level. Moreover, given the prevalence of non-ergodicity in biological and social sciences (*29*), these methods complement mass-univariate analyses and should be routinely used to ensure consistency between group and individual correlations. Only a few studies have, so far, pursued multivariate classification of functional and structural neuroimaging data to predict CBT response in MDD (*10*, *15*–*17*). A major limitation of these studies is the lack of mechanistic interpretability of results. Although it is arguable that any actionable biomarker informing clinical decision-making is useful, biomarkers that are embedded in disease pathophysiology are superior as they provide additional advantages. First, mechanistic biomarkers afford important insights into key pathophysiologic processes and may thus help replace the current symptom-based nosology of mental disorders with brain-based diagnostic categories. This fits in well with recent research initiatives such as the Research Domain Criteria framework. Second, they hold the potential to inform development of novel brain-based treatments by uncovering previously unidentified neural therapeutic targets. Third, they facilitate recruitment of more homogeneous samples into clinical trials so that the efficacy of newly developed therapeutic interventions can be adequately tested without confounding factors biasing the results. For these reasons, it is therefore imperative to select mechanistically interpretable fMRI features and to quantify their predictive power.

Although, in depression research, it is a common practice to define clinical response as a ≥50% reduction from baseline depression severity to endpoint, this is somewhat arbitrary. According to this criterion, responders may still be left with substantial residual symptoms despite symptom reduction. Conversely, nonresponders may be in remission although the percentage change in their depression severity score is less than 50%. Furthermore, clinical response to treatment occurs on a continuum from better to worse, and categorical classifications ignore this heterogeneity of treatment effects. Taking this into account, a biomarker providing clinicians with an estimate of the likelihood that a patient will benefit from a given treatment or of the magnitude of his/her future symptomatic change will better inform clinical judgment. In this study, we show that activity encoding the weighted RPE yields a probabilistic estimate of posttreatment clinical response, which is significantly and linearly correlated with observed symptomatic improvement.

We trained our classifier on neural features denoting a putative disease process such as biased information acquisition and processing during RL, which has been shown to be impaired not only in MDD but also in other psychiatric disorders. Therefore, it is possible that our findings may, in fact, represent transdiagnostic neural biomarker predictors of CBT response. So far, we have contended that selection of theory-driven neural features ensures model’s pathophysiological plausibility and contributes to advancing our understanding of brain pathology. However, it also has the critical additional benefit of preventing classification from relying on neural noise or nonmeaningful neural activity. The path toward development and validation of clinically viable and reliable neuroimaging predictors of CBT response in MDD involves a number of sequential steps ranging from model development to testing a model’s performance on diverse, large-scale populations. According to this framework, our study is the initial stage of model development. Future work should seek to replicate our findings in larger and independent samples. The lack of a completely independent test set in our study precludes any definitive conclusion regarding the predictive nature of observed imaging biomarkers. Moreover, since we obtained the contrast images encoding the weighted RPE by fitting a computational model to the entire sample, predictions at the individual level are not completely independent.

In conclusion, in this study, we have provided evidence supporting utility and feasibility of a neurocomputational approach to treatment response classification in depression and, more generally, in mental health research. In particular, we have shown that computational and neural correlates of probabilistic RL enable early discrimination of treatment response to cCBT in depression.

## Supplementary Material

http://advances.sciencemag.org/cgi/content/full/5/7/eaav4962/DC1

Download PDF

## SUPPLEMENTARY MATERIALS

Supplementary material for this article is available at http://advances.sciencemag.org/cgi/content/full/5/7/eaav4962/DC1 fMRI analysis of posttreatment data Fig. S1. Posttreatment activity change.
