# Supplementary material for: Neural correlates of weighted reward prediction error during reinforcement learning classify response to cognitive behavioral therapy in depression
Source: Sci Adv. 2019 Jul 31;5(7):eaav4962. doi: 10.1126/sciadv.aav4962 (PMC6669013; doi:10.1126/sciadv.aav4962)
Supplement: http://advances.sciencemag.org/cgi/content/full/5/7/eaav4962/DC1 [file supp_5_7_eaav4962__index.html]

Science Advances | Science AdvancesAAASSearchScience AdvancesMenu

## Supplementary Materials

**This PDF file includes:**

- fMRI analysis of posttreatment data
- Fig. S1. Posttreatment activity change.

Download PDF

**Files in this Data Supplement:**

- Adobe PDF - aav4962\_SM.pdf
