# Supplementary material for: Neural correlates of weighted reward prediction error during reinforcement learning classify response to cognitive behavioral therapy in depression
Source: Sci Adv. 2019 Jul 31;5(7):eaav4962. doi: 10.1126/sciadv.aav4962 (PMC6669013; doi:10.1126/sciadv.aav4962)
Supplement: Download PDF [file aav4962_SM.pdf]

Science Advances
AAAS

advances.sciencemag.org/cgi/content/full/5/7/eaav4962/DC1

Supplementary Materials for

Neural correlates of weighted reward prediction error during reinforcement learning classify response to cognitive behavioral therapy in depression

Filippo Queirazza*, Elsa Fouragnan, J. Douglas Steele, Jonathan Cavanagh, Marios G. Philiastides*

*Corresponding author. Email: filippo.queirazza@glasgow.ac.uk (F.Q.); marios.philiastides@glasgow.ac.uk (M.G.P.)

Published 31 July 2019, *Sci. Adv.* **5**, eaav4962 (2019)
DOI: 10.1126/sciadv.aav4962

This PDF file includes:

fMRI analysis of posttreatment data
Fig. S1. Posttreatment activity change.

SUPPLEMENTARY MATERIALS

fMRI analysis of posttreatment data

Although unfortunately owing to the number of dropouts and ensuing unbalanced design we were unable to perform a mixed effect analysis of variance to test for between-session and between-group effects, we still checked for a relationship between change in brain activity and symptomatic improvement. Although this was not strictly speaking a mediation analysis, its purpose was to clarify whether neural activity of putative biomarkers is being modulated by cCBT and which role it plays in the context of clinical recovery. To this end, we initially fitted the best fitting computational model to the post-treatment behavioural data and used the model fits to run 1st level fMRI analysis on post-treatment data with same design matrix and contrasts as for pre-treatment data. Subsequent to this, we used the two statistically significant clusters from the pre-treatment 2nd level unpaired t-test as masks to retrieve the cluster-wise average beta weights from the spatially normalised post-treatment contrast images encoding the weighted RPE. Finally we estimated the cluster-wise difference between pre- and post-treatment beta weights for each subject and correlated these with magnitude of symptomatic change using robust 20% bend correlation.

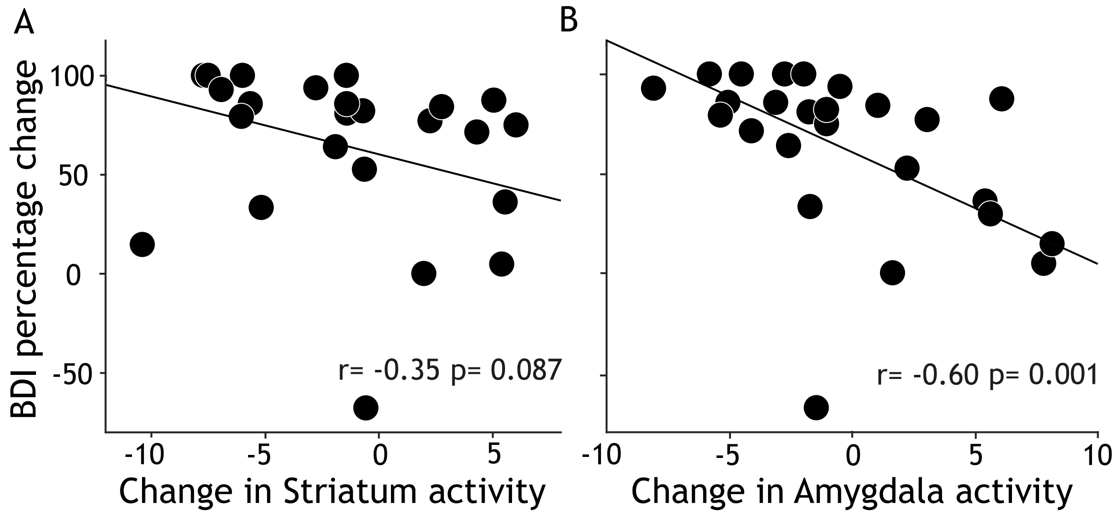

Fig. S1. Posttreatment activity change. Scatterplot (n=25, 18 responders and 7 non-responders) showing robust linear correlation between percentage change in post-treatment BDI scores and subject-specific change (post-treatment minus pre-treatment) in average parameter estimates extracted from two clusters pertaining to the right striatum **(A)** and right amygdala **(B)**.
